# E-Readers and Visual Fatigue

**DOI:** 10.1371/journal.pone.0083676

**Published:** 2013-12-27

**Authors:** Simone Benedetto, Véronique Drai-Zerbib, Marco Pedrotti, Geoffrey Tissier, Thierry Baccino

**Affiliations:** 1 CHArt/LUTIN, Université Paris 8, Paris, France; 2 CHArt/LUTIN, Université Paris 6, Paris, France; University of Leicester, United Kingdom

## Abstract

The mass digitization of books is changing the way information is created, disseminated and displayed. Electronic book readers (e-readers) generally refer to two main display technologies: the electronic ink (E-ink) and the liquid crystal display (LCD). Both technologies have advantages and disadvantages, but the question whether one or the other triggers less visual fatigue is still open. The aim of the present research was to study the effects of the display technology on visual fatigue. To this end, participants performed a longitudinal study in which two last generation e-readers (LCD, E-ink) and paper book were tested in three different prolonged reading sessions separated by - on average - ten days. Results from both objective (Blinks per second) and subjective (Visual Fatigue Scale) measures suggested that reading on the LCD (Kindle Fire HD) triggers higher visual fatigue with respect to both the E-ink (Kindle Paperwhite) and the paper book. The absence of differences between E-ink and paper suggests that, concerning visual fatigue, the E-ink is indeed very similar to the paper.

## Introduction

Reading behavior has been investigated by psychologists for several decades, some of them focusing on low-level processing of words such as visibility [1] or legibility [2] and others on comprehension levels [3], [4], [5]. Although researchers generally studied them separately, these three theoretical levels are very practical for delimiting reading [6]. Usually, the visibility processing (i.e. distinguishing a visual signal from the background) is not a matter of interest in reading since everyone assumes that visual factors are generally fitted in reading experiments. This assumption would be true if any linguistic material was presented on the same support. In the real world this cannot be true, and the use of computer displays for presenting linguistic material may involve a large variability. For example, it has been shown that the display polarity (negative/positive polarity) [7] or the refresh rate [8], [9] might affect vision during reading.

In the era of digitalization, nothing remains untouched and paper books are no exception. Electronic books (e-books) are changing the way information is created, disseminated and displayed. Although e-books are usually displayed on dedicated e-book readers (e-readers), almost any electronic device equipped with reading software can be used to read an e-book. With respect to traditional books, the advantages as well as the disadvantages of digital books are many. E-books are hypertexts that allow carrying an entire library within a small space, they are cheaper (about 50–60% lower than print), more environmental friendly, and they share higher levels of text personalization (e.g. font size, font type, color and luminance). At the same time they show issues related to piracy (e-books are easier to copy) and are less emotionally involving (e.g. lack of tactile and olfactory feedback).

E- readers generally refer to two main display technologies: the electronic ink (E-ink) and the liquid crystal display (LCD). The E-ink (i.e. electronic ink or electronic paper) is designed to reproduce the appearance of ink on paper. With respect to LCD, the main advantages of E-ink display are better readability of their screens - especially in bright sunlight - and longer battery life. While E-ink readers do not allow colors and are limited for reading, LCD e-readers are usually tablets, which means they are not just a replacement for a book, rather multifunctional devices, which can be used for communication, organization or leisure activities [10]. LCD tablets have faster screens capable of higher refresh rates and are more suitable for interaction. Some last generation E-ink displays, like the Kindle Paperwhite, offer a reading experience in all lighting conditions, from bright sunlight to bedtime reading, guiding light towards the surface of the e-ink display from above.

In forums about e-readers there are many statements about the advantages and disadvantages of these displays and their term of comparison is usually the paper, which is still the most used support for reading. According to Siegenthaler et al. [10], the discussion whether E-ink or LCD is better for reading is emotional, and scientific evidence is quite sparse. In fact, just few studies are focused on reading behavior and even less deal with visual fatigue. Moreover, the results of these studies are device-dependent, and the rapid technological advancement of these supports turns recent results out of date quite quickly.

Concerning reading behavior, Shen et al. [11] found E-ink reader (Sony e-reader) to have higher search accuracy with respect to LCD (Kolin e-reader). Siegenthaler et al. [12], found no differences between the same E-ink device (Sony e-reader) and LCD (iPad 1^st^ generation), as confirmed by both subjective (VFS - [13]) and objective measures (eye and reading performance measures). Siegenthaler et al. [10] showed that iPad 1^st^ generation, under special artificial light conditions, may even provide better legibility than Sony e-reader. Siegenthaler et al. [14], comparing five E-ink displays and a paper book, found that reading behavior on e-readers is very similar to the reading behavior on print (i.e. no differences in reading speed and regressive saccades), and that E-ink may even provide better legibility than paper. Zambarbieri & Carniglia [15] found no differences in reading behavior between paper book, iPad 1^st^ generation and E-ink (Kindle DX).

The aim of the present research was to study the effects of the display technology on visual fatigue using prolonged reading sessions [16]. According to the International Classification of Diseases (ICD-10) of the World Health Organization (WHO), visual fatigue - also called visual strain - is classified as a subjective visual disturbance (H53.1), manifested by a degree of visual discomfort typically occurring after some kind of prolonged visual activity, and characterized by fatigue, pain around the eyes, blurred vision or headache.

In this framework, the need to empirically evaluate visual fatigue on e-readers and paper is getting more and more important. However, only few studies have focused on visual fatigue [11], [12], [17], [18]. Kang et al. [17] found LCD (Kolin e-reader) to trigger higher visual fatigue than paper book as well as lower reading performance. Lee et al. [18] showed that Sony e-reader triggers shorter search times and higher accuracy with respect to LCD (Kolin e-reader), whereas no differences were found as to visual strain.

In our experiment, participants performed a longitudinal study in which two last generation e-readers (LCD, E-ink) and paper book were tested in three different reading sessions separated by - on average - ten days. The experiment consisted of prolonged reading (>1 hour) on each device while eye data were recorded. Subjective and objective visual fatigue measures were collected at the beginning and at the end of each reading session. Variables such as font size, typeface and number of words per page were not manipulated and were kept constant during the whole experiment, as well as across the three devices [17], [19]. If reading on E-ink, LCD, and print is similar, then no differences in objective and subjective measures should be found.

## Materials and Methods

### Participants

Twelve participants (5 males, mean age = 27 years, SD = 4) volunteered for the experiment. All of them had no previous experience with e-readers, had normal or corrected-to-normal vision, and were naïve as to the aims and the expected outcomes of the experiment. Participants gave written informed consent before participation. The study was performed in keeping with the declaration of Helsinki. The protocol was approved by the French National Board of Informatics and Freedom.

### Apparatus

Eye data were recorded with a 30 Hz infrared video-based eye tracker (SMI Eye Tracking Glasses - ETG). In order to ensure the best tracking quality, calibration was made for each participant at the beginning of each reading trial and further checked at the end of each one. Measurements were taken under constant artificial illumination. As assessed by a digital light meter sensor (Extech 403125; Extech Instruments, Nashua, NH) placed on the participants’ forehead at 60 cm from the reading device, the amount of light incident on that area totaled 54 lx. This measurement did not vary among the three reading devices.

### Stimuli

**Figure 1:**
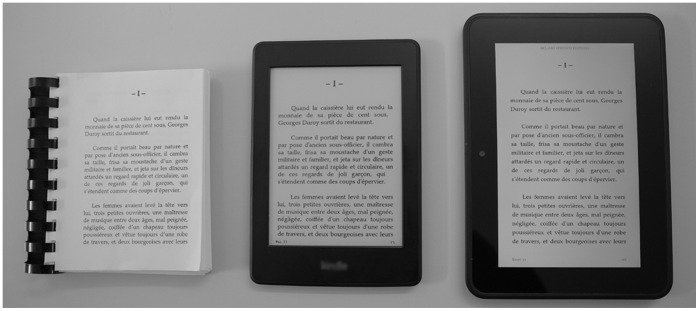
The three reading devices used in this study: the paper book, the Kindle Paperwhite and the Kindle Fire HD (from left to right).

Three different reading devices were chosen: two last generation e-readers (of the same brand) and a paper book (Fig. 1). Concerning e-readers, the selection criteria were a) the display technology (both LCD and E-ink), and b) the ranking based on users’ reviews (http://ebook-reader-review.toptenreviews.com). According to these criteria a Kindle Fire HD (LCD display) and a Kindle Paperwhite (E-ink display) were employed. Specifications of the three reading devices are shown in Table 1.

**Table 1:** Device specifications.

Reading device	Support size	Support type	Resolution
Kindle Fire HD	7″	HD LCD	216 ppi
Kindle Paperwhite	6″	Paperwhite display	212 ppi
Paper book	6″	Paper	300 dpi

Since the text displayed on each support had to be identical across the three devices (same page size, font size, typeface and number of words per page), regulations were made accordingly. As to the paper book, each single page was edited using a word processor, and a spiral binding was used to allow pages to lay flat, improving page turn (see Fig. 1).

In order to allow comparison among the devices, we tried to find a compromise between the level of luminance of the devices and their readability. To this end, the level of luminance emitted by the e-readers’ displays was adjusted at the beginning of the experiment. Obviously, since the paper reflects but does not emit light directly, the luminance of the paper book could not be manipulated. The Michelson definition of contrast [20] was used to determine the actual contrast ratios [C = (L_max_ − L_min_)/(L_max_+L_min_)] where C = contrast, L_max_ = maximal luminance, L_min_ = minimal luminance. We measured luminance for black (minimal luminance) and white (maximal luminance) displays for the two reading devices. As assessed by a digital luminance meter for contact measurements (Mavo-Monitor; Gossen, Germany), Michelson contrast ratios were as follows: Kindle Fire HD (LCD): 0,99 (L_max_: 20 cd/m^2^; L_min_: 0,05 cd/m^2^); Kindle Paperwhite (E-ink): 0,91 (L_max_: 2,10 cd/m^2^; L_min_: 0,10 cd/m^2^).

We then measured the total amount of light (emitted plus reflected) by each device with a digital luminance meter (Konica Minolta LS-110; Tokyo, Japan) placed at 60 cm from the reading device at the exact lighting settings and eye level used during data acquisition. The Michelson ratios were as follows: Kindle Fire HD (LCD): 0,96 (L_max_: 27,77 cd/m^2^; L_min_: 0,58 cd/m^2^); Kindle Paperwhite (E-ink): 0,77 (L_max_: 11,27 cd/m^2^; L_min_: 1,44 cd/m^2^); Paper book: 0,90 (L_max_: 16,42 cd/m^2^; L_min_: 0,86 cd/m^2^).

### Experimental Design and Procedure

A longitudinal full within-subjects design was employed. Each participant read on each of the three reading supports. The sequence of the reading devices was randomized to control for order effects. The experiment took place in three sessions separated by ten days on average (Session 1, Session 2, Session 3). Reading sessions took place at the same time of the day. Text material was a novel [21] in French language, i.e. the mother tongue of all the participants. For each reading session a different part of the book was employed (see Table 2). Since adults - on average - read prose text at 250 to 300 words per minute [22], [23] we expected people to complete each reading session in about 70 min.

**Table 2:** Number of characters (without spacing) and number of words for each reading session.

Readingsession	Chapters	Number ofcharacters	Number ofwords
1	I–II–III	75341	15943
2	IV–V	78042	17037
3	VI–VII	75473	16329

**Figure 2:**
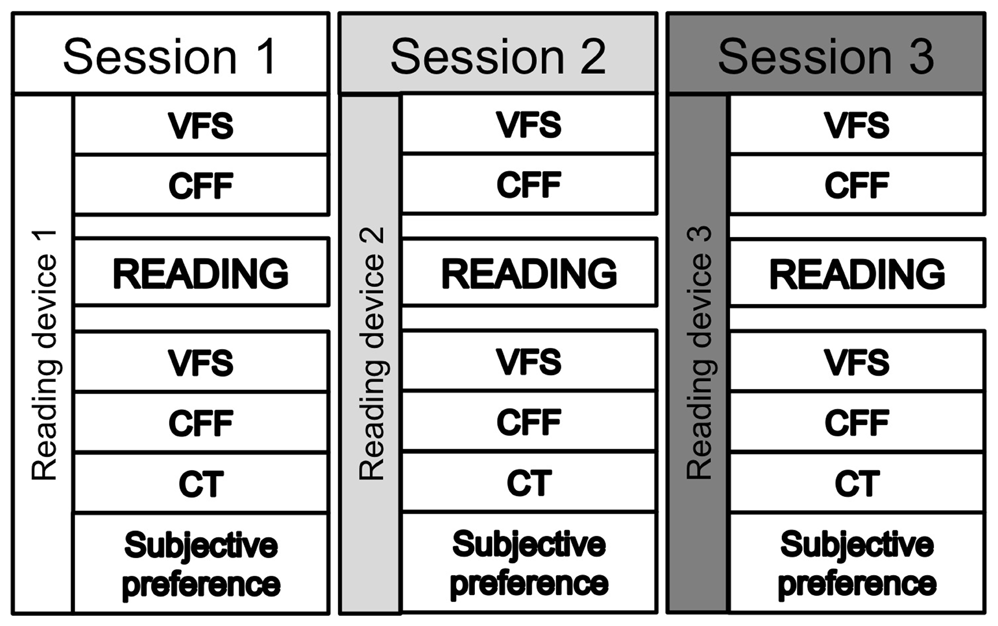
Experimental procedure: schematic representation. VFS = Visual Fatigue Scale; CFF = Critical Flicker Fusion; CT = Comprehension Test.

The test was performed in a controlled and standardized room at LUTIN - Paris (www.lutin-userlab.fr). A schematic representation of the procedure is provided in Figure 2.

After giving written consent, participants familiarized themselves with the reading device. Each experimental session started with the subjective visual fatigue scale (VFS - [13]), and was followed by the Critical Flicker Fusion test (CFF). After that, participants sat on a comfortable chair at a fixed distance of approximately 60 cm from the reading support (placed on a tailor-made bookrest allowing a 45° reading angle) and the eye tracker was calibrated.

Participants were then required to silently read the selected part of the novel (see Table 2) on one of the randomly assigned supports, while their eye data were recorded. At the end of each reading session, participants underwent the VFS and the CFF for the second time. After that, a comprehension test (CT) was administered in order to verify that participants had effectively read the book, together with a subjective preference scale [5]. The CT consisted of 5 questions for each of the three reading sessions selected from http://colleges.ac-rouen.fr/abaquesne/activites/francais/belam/qcmbelam.htm.

### Dependent Variables

#### CFF (Critical Flicker Frequency)

The flicker fusion is the visual phenomenon in which a repetitively presented stimulus (flickering stimulus) appears as a single continuous stimulus. A fall in *CFF* values reflects a drop in the sensory perception function, attributable to a decrease in alertness [24]. As to visual fatigue, there are controversies related to the reliability of this measure. Within similar studies, to the authors’ knowledge just one study [17] was able to detect changes in visual fatigue whereas others were not [11], [18]. With the aim to verify the soundness of the CFF, a staircase method collecting three ascending and three descending presentations (with 1 Hz steps) in an alternating order was used to find the thresholds. For the ascending trials, participants were required to indicate when the light appeared to transition from flickering to continuous by pressing a button. Conversely, for the descending trials, they were required to press the button when the light appeared to transition from continuous to flickering. The *CFF* was measured in Hz at the beginning and at the end of each reading session.

#### Eye blink

The eye blink, the rapid closing and reopening of the eyelid, is well known indicator of visual fatigue [25], [26], [27].

A large body of literature suggests that blinks decrease during reading (e.g. [28]), and even more when reading on backlit video display terminals (VDT) [29], [30], [31], [32], [33], [34]. According to Blehm [30], such a reduction contributes to a poor tear film quality and temporarily stresses the cornea (producing increased corneal exposure), causing dry eye. The dry eye is one of the most common symptoms of the Computer Vision Syndrome (CVS), which is the combination of eye and vision problems associated with the prolonged use of video terminals [32].

With the aim of verifying the hypothesis that reading on backlit display decreases the number of blinks with respect to hard copy material, the number of eye blinks per second (*BPS*) was chosen as a dependent variable. *BPS* was calculated as the quotient of the total amount of eye blinks that occur in each reading session divided by the duration (in seconds) of each reading session.

#### Visual Fatigue Scale (VFS)

A rating scale of visual fatigue (*VFS* - [13]) was administered at the beginning and at the end of each reading session. It consisted of six items: 1) I have difficulties in seeing; 2) I have a strange feeling around the eyes; 3) My eyes feel tired; 4) I feel numb; 5) I have a headache; 6) I feel dizzy looking at the screen. Each item was rated on a 10-point Likert scale.

#### Subjective preference

Similarly to Siegenthaler et al. [14], subjective preference was judged on a 7-point Likert scale for each reading device.

## Results

The significance level α was set at.05 for all statistical analyses. Cardinal variables (i.e. *CFF*, *BPS*) were analyzed with a repeated measures analysis of variance (rmANOVA), and *p* values were adjusted following a Greenhouse-Geisser correction [35]. Ordinal variables (i.e. *VFS*, *Subjective preference*) were analyzed with a Friedman’s ANOVA [36], while the Wilcoxon paired-sample test method [37] was used for planned comparisons (a Bonferroni correction [38] was applied). Means and standard deviations for each of the dependent variables are reported in Table 3.

**Table 3:** Means and standard deviations (italic) for each of the dependent variables.

Dependent Variable		Reading Device		
		LCD	E-ink	Paper
**CFF** (Hz)	Before	41,60 (*1,66*)	41,54 (*1,65*)	41,82 (*1,70*)
	After	40,65 (*1,48*)	41(*1,76*)	41,28 (*1,44*)
**VFS** (1–10)	Before	1,76 (*0,62*)	1,85 (*0,89*)	1,79 (*1*)
	After	3,36 (*1,55*)	2,90 (*1,65*)	2,44 (*1,58*)
**BPS** (blinks/second)		0,43 (*0,19*)	0,61 (*0,25*)	0,61 (*0,32*)
**Subjective Preference**(1–7)		3,55 (*1,44*)	4,45 (*1,88*)	6,64 (*0,64*)

The first requirement for proceeding further into the analysis was to make sure that participants had effectively read and understood the book. This was validated by the absence of wrong answers to the CT. The second requirement consisted in verifying that participants spent at least one hour on each reading session: independently from the support, participants read on average 73 min (SD 10 min). For reference only, an analysis on reading speed was conducted and no significant differences were found neither between the reading supports nor the reading sessions (all *F*s n.s.).

Two variables underwent a measurement before and after reading: the *CFF* and the *VFS*.

**Figure 3:**
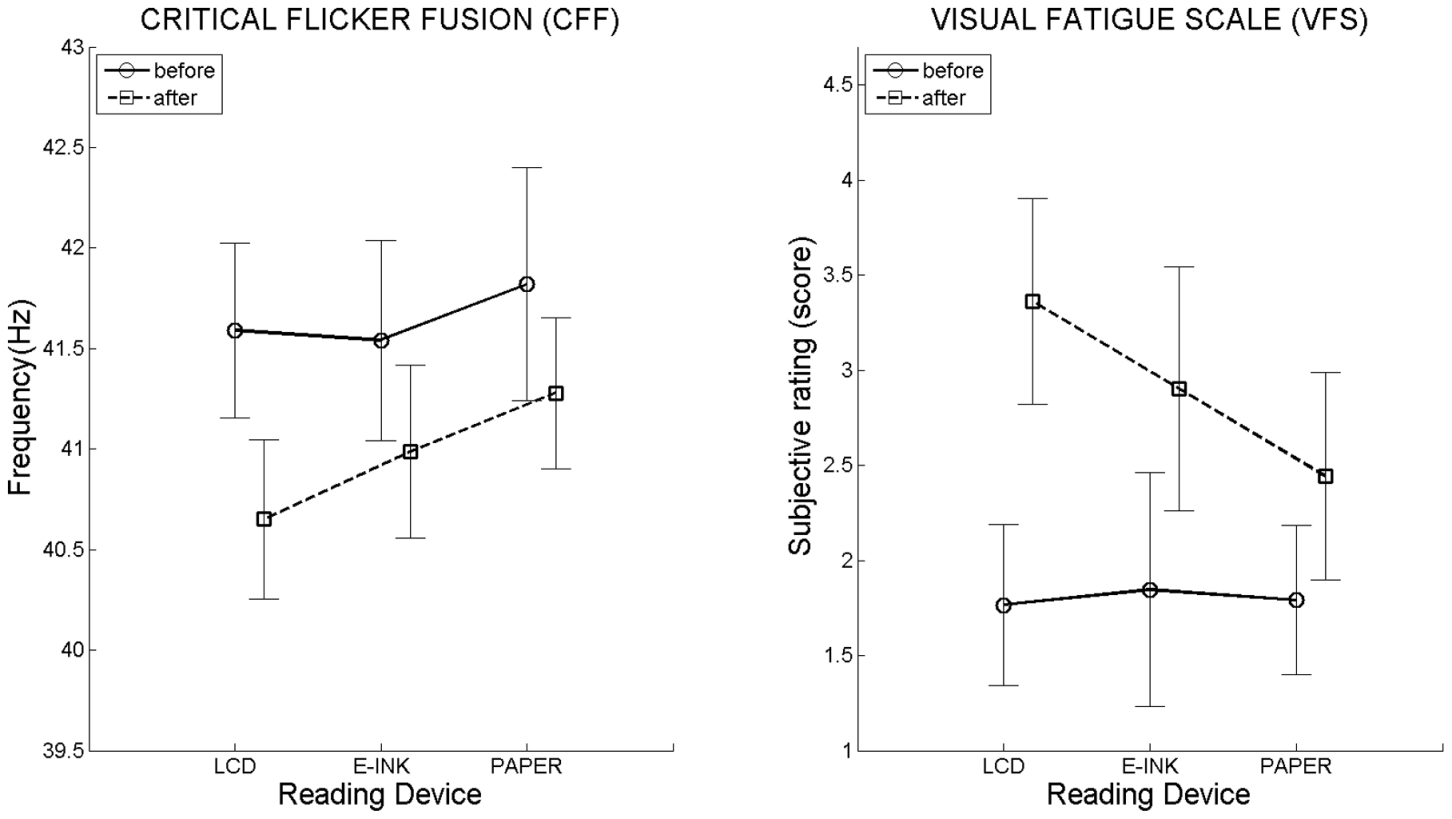
CFF (left) and VFS (right) before and after reading for each reading device. Vertical bars denote 95% confidence intervals calculated using Morey’ s correction [39], [40]. N = 12.

As to the *CFF*, a time effect was found (*F*(1, 11) = 15,91, p<.005 *η^2^_p_* = .59, Figure 3-left), revealing a significant drop in sensory perception after reading, independently from the device. Furthermore, no device effect and no interactions between device and time were found.

With regard to the VFS, a main effect was found (*X^2^_r_* (5, 12) = 30.83, *p*<.001, Figure 3-right). Higher scores were found after reading on the LCD (*Z* = 2.93, *p*<.01, *r* = .60), whereas no differences were found for the E-ink (*Z* = 2.12, n.s.), nor for the Paper book (*Z* = 2, n.s.).

**Figure 4:**
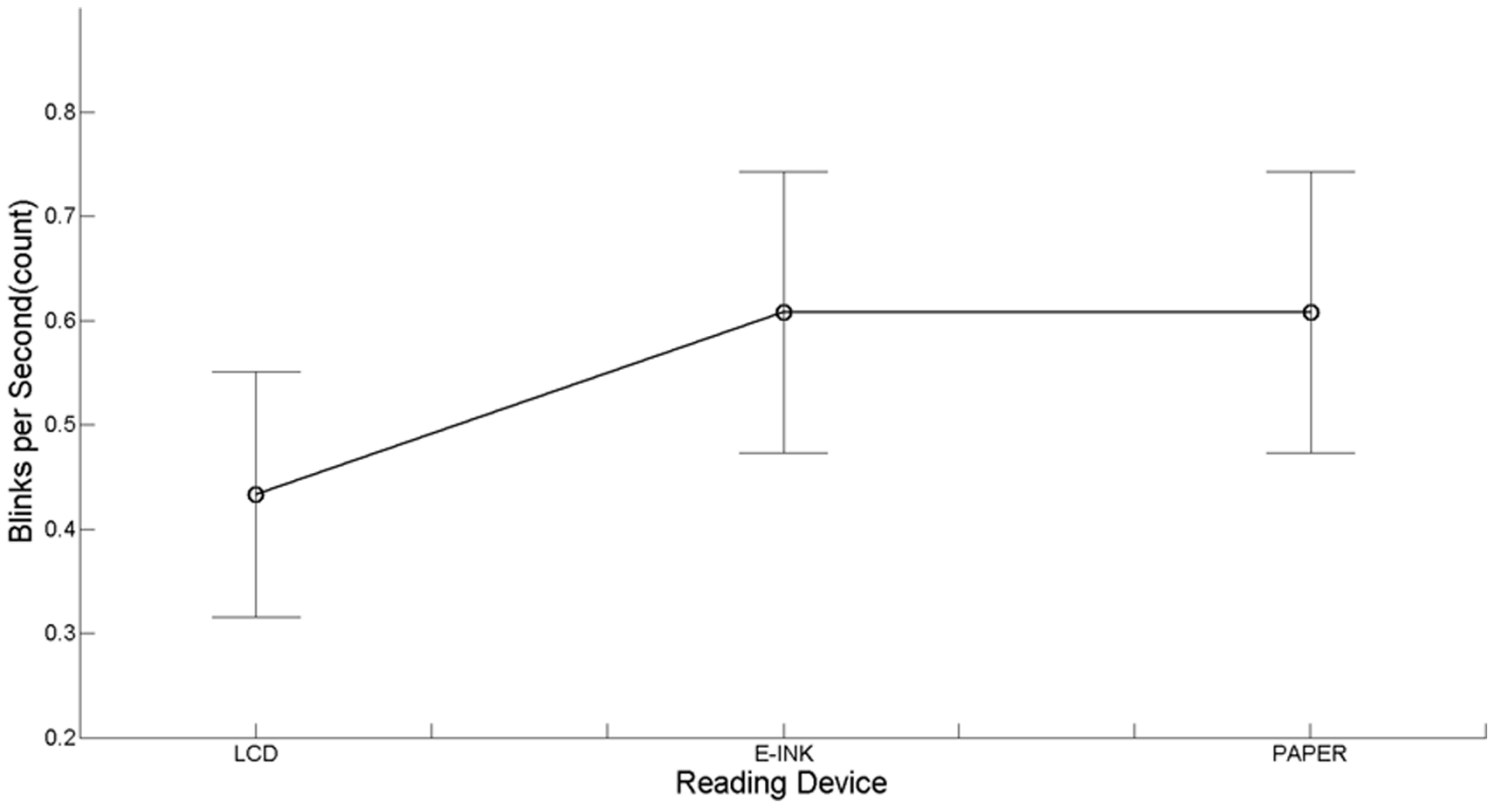
BPS for each reading device. Vertical bars denote 95% confidence intervals calculated using Morey’ s correction [39], [40]. N = 12.

Concerning the number of eye blinks per second (*BPS*), since the Shapiro-Wilk’s test [41] revealed data to be not normally distributed, a Box-Cox transformation [42] was applied. A main effect was found (*F*(2, 22) = 4.17, *p*<.05, *η^2^_p_* = .27, Figure 4). Planned contrasts between LCD and E-ink (*F*(1, 11) = 6.30, *p*<.05, *η^2^_p_* = .36) and between LCD and Paper (*F*(1, 11) = 6.59, *p*<.05, *η^2^_p_* = .38) showed that reading on LCD significantly decreases the number of blinks with respect to other devices. Furthermore, the non-significant planned contrast between Paper and E-ink (*F*(1, 11) = .06, n.s.), revealed that both the devices generate a very similar blink behavior.

With the aim of verifying whether our results are attributable to the higher level of luminance emitted by the LCD, an analysis of average pupil size (*APS*) was carried out. Since the human pupil primarily *constricts* as luminance increases [43], reduced *APS* was expected for the LCD. A main effect was found (*F*(2, 22) = 11.92, *p*<.001, *η^2^_p_* = .52). Planned contrasts between LCD and E-ink (*F*(1, 11) = 27.12, *p*<.001, *η^2^_p_* = .71) and between LCD and Paper (*F*(1, 11) = 9.15, *p*<.05, *η^2^_p_* = .45) showed that the higher level of luminance emitted by the LCD (see Materials and Methods) reduces the size of the pupil with respect to the other devices. Furthermore, the non-significant planned contrast between Paper and E-ink (*F*(1, 11) = 1.88, n.s.) revealed that reading on these devices leads to similar tonic pupil diameter.

**Figure 5:**
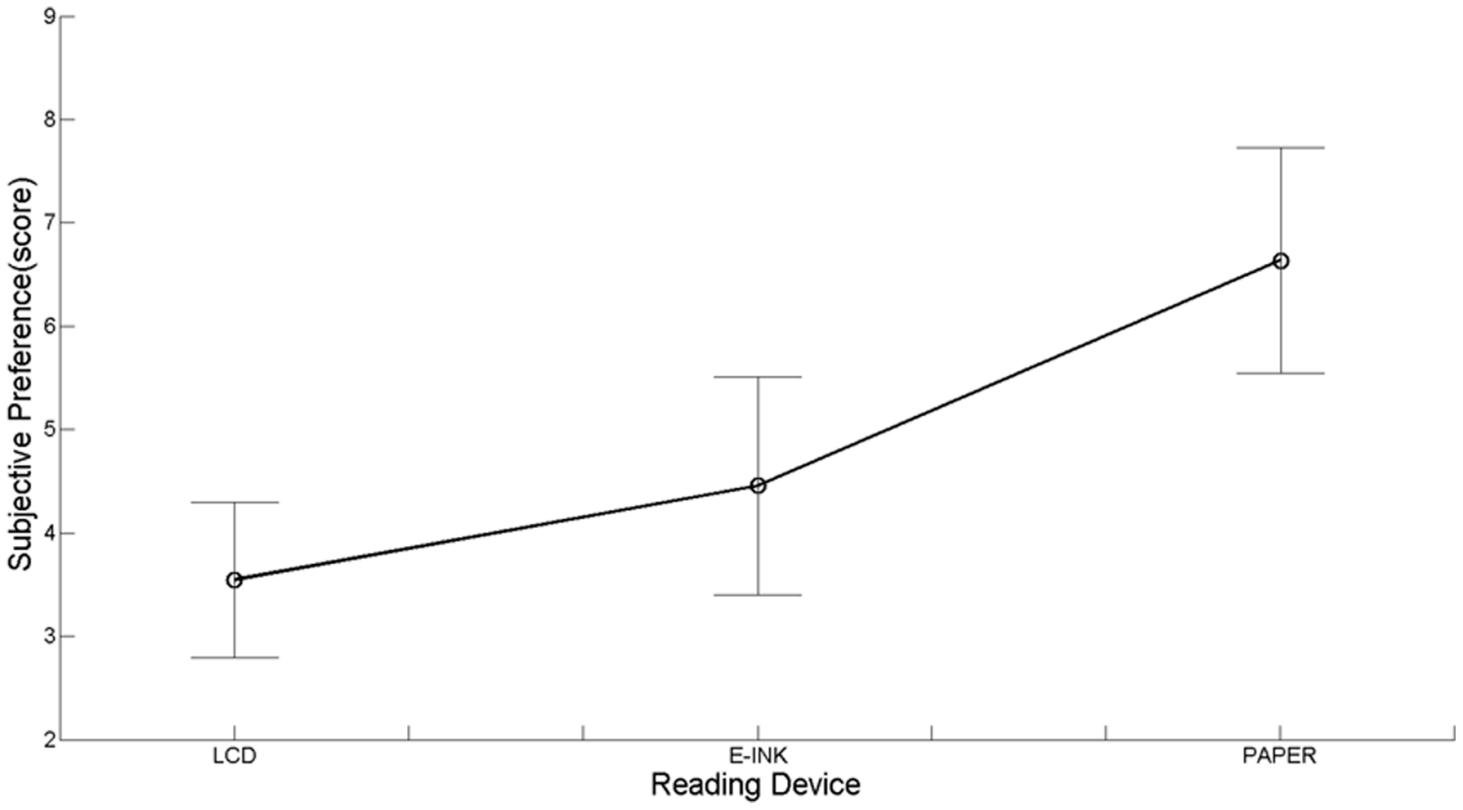
Subjective preference for each reading device. Vertical bars denote 95% confidence intervals calculated using Morey’ s correction [39], [40]. N = 12.

As to *Subjective preference,* a main effect was found (*X^2^_r_* (2,12) = 13.35, *p*<.005, Figure 5). Planned contrasts between LCD and Paper (*Z* = 2.93, *p*<.01, *r* = .60) and between Paper and E-ink (*Z* = 2.49, *p*<.05, *r* = .51), indicated a preference for the paper book. The planned contrast between LCD and E-ink (*Z* = 1.52, n.s.), revealed no difference between the two devices in terms of personal preference.

## Discussion

The aim of the present study was to compare prolonged reading on three different supports regarding their effects on visual fatigue. Likewise Kang et al. [17], and Chang et al. [19], variables such as font size, typeface and number of words per page were not manipulated and were kept constant across the three devices for the entire reading sessions. Subjective measures (*VFS*) suggested that prolonged reading on the LCD (Kindle Fire HD) triggers higher visual fatigue with respect to the E-ink (Kindle Paperwhite) and the paper book. Concerning objective measures (*BPS* and *CFF*), contrasting results were found.

As to *CFF*, results revealed a significant drop in sensory perception after reading independently from the device, thus failing to show significant differences among the three reading supports. On the one hand, these results are in line with previous studies employing the *CFF* for similar purposes, which did not succeed in finding differences between paper book, E-ink and LCD [11] and between E-ink and LCD [18]. On the other hand, although our experimental plan has some similarities with that of Kang et al. [17], where the task consisted of reading novelettes for 40 to 60 min, our results are contrasting. These authors found a significant difference between the paper book and the LCD, namely a larger *CFF* reduction when reading on the LCD. In the present study, we could not replicate this finding.

With regard to *BPS*, experimental evidence indicated that reading on the LCD leads to a larger decrease in the number of blinks, with respect to the other supports. This result is in line with a large number of studies on CVS (for a review see [32]), where the use of backlit displays is usually associated with a decreased frequency of blinking and an increased rate of tear evaporation, each of which contributes to dry eyes. In fact, prolonged display exposure contributes to incomplete blinking provoking tear film instability [44], which is one of the main factors for visual fatigue on VDT [45].

In contrast with previous studies, where no differences in terms of perceived visual fatigue (*VFS*) were found between LCD, E-ink and paper book [2] and LCD and E-ink [18], our results showed that participants felt visually fatigued only when reading on the LCD. Such a finding might be attributable to the longer reading sessions employed in our study (on average 73 min, SD 10 min), with respect to previous studies [11], [18].

Finally, results on *Subjective preference* suggest that participants with no experience with e-readers prefer paper books. The overall belief that digital reading media reduce the pleasure of reading could be cultural rather than cognitive [46]. Moreover, since reading habit for paper books is normally fixed in childhood [17], it’s quite obvious that people prefer paper books rather than e-books.

In conclusion, our results might be imputable to the higher level of luminance emitted by the LCD (see Materials and Methods). With respect to the paper book and the E-ink, reading on the LCD reduces the size of the pupil (*APS*) and the frequency of eye blink (*BPS*), and increases the perceived visual fatigue (*VFS*).

Although the Kindle Fire HD adopts a last generation LCD with IPS (in-plane switching) technology, advanced polarizing filter, and anti-glare technology, the issues related to backlight technology are still present. In contrast to LCD-displays, which have been associated with impaired reading performance [14] and higher visual fatigue [17], results on E-ink displays are encouraging.

Since visual discomfort and related symptoms occurring in VDT workers have been recognized as a growing health problem [32], we believe that the growing spread of e-readers should be taken into account as well. Although the aim of this study was to make an up-to-date comparison of reading devices concerning their effects on visual fatigue, it should be emphasized that comparisons with previous studies, employing older display technologies, have some limits. The use of reading devices as independent variables clearly leads to device-dependent results.

Future studies will include the manipulation of the length of the reading sessions, the luminance levels of the displays, and the study of binocular vision on prolonged reading [47].
